# Annotations

**Published:** 1889-11-16

**Authors:** 


### Annotations.

**A City of Cesspools.**

It will hardly be believed that such a city exists in England to-day, much less that the inhabitants at the recent municipal elections have decided by their deliberate choice, not only that the cesspools and contaminated wells shall remain, but that further cesspools shall be constructed. The surprise will be increased when it is stated that this unfortunate town is none other than the cathedral city of Chichester, often called the City of Gardens, from the fact that nearly every house has a garden, and that it comprehends within its area fields and open country. In 1865, 1867, and 1879 sanitarians of the eminence of Dr. Seaton, Mr. Hawkesley, and Dr. Airey, as representatives of the Government, and therefore impartial and disinterested observers, declared the whole site of Chichester to be honeycombed with cesspools; that the gravel bed beneath is charged with cesspool matter; that the city must be open to mistrust until some system of internal drainage is adopted; that the cesspools are often in dangerously close relation with the wells from which a large proportion of the drinking water is still supplied, very many houses being entirely dependent on wells for their water supply; and that in respect of drainage there is apparent community of unwholesome conditions. Dr. Airey further states he saw places where milk-cans were being washed with water drawn from a well which could not be free from pollution, seeing that there was a privy pit only eight yards off in one direction, and a cesspool only seven yards off in another. It is a natural consequence of this state of affairs that the mortality of Chichester is 2·5 above the average death-rate of the adjacent sanitary districts of Sussex, and that the mortality from consumption exceeds that of the whole of England by ·53 per 1,000. The number of deaths from typhoid fever are nearly double those of England, and nearly five times greater than the average of the adjacent sanitary districts. These facts are notorious, they have been recognised for more than a quarter of a century, and their continuance and extension have now been voted by a majority of the inhabitants in every part of the city. The Town Council, in a report dated September 2 last, recommended the adoption of a new scheme of drainage to remedy all these evils with as little delay as possible, but the ratepayers have rejected the drainage proposals, and have accepted the views of a local medical practitioner, who signed an address advocating the digging of more cesspools so constructed as to allow the sewage to percolate through the sides and bottoms into the surrounding soil, with the object, apparently, of saving the householders the expense of emptying them. It is difficult to decide whether the reckless parsimony of the ratepayers, or the astounding ignorance of the sanitary law displayed by the local doctor, who received more votes than any other candidate, deserve the severest censure. The Church, too, stands self-condemned; for both the Bishop and the Dean held aloof, and neither raised a finger to save their flocks, not even the innocent children, from a very real danger, which, if much longer continued, will reduce Chichester to a state of desolation arising from the destruction of its inhabitants by epidemic disease. The Town Council showed that Chichester can be saved from death by an expenditure of £15,435, but the ratepayers will not provide this paltry sum. What will happen if cholera breaks out in the summer or autumn it is not difficult to foretell, and meanwhile those inhabitants who have any intelligence, or any regard for their own safety and that of their wives and families, should proceed by mandamus to enforce the law as to cesspools (Public Health Act, sec. 47, 3). This will force a very large expenditure on the landlords and builders, and an annual charge probably far in excess of any addition to the rates which a proper system of drainage would entail. It is necessary, however, to teach people who behave as those responsible for the present fatally dangerous condition of Chichester have behaved, that minorities have their rights, and one of them is the protection of themselves and those nearest and dearest to them from the perpetual risk of a lingering and painful death. Nothing more disgraceful to the intelligence and proper feeling of a whole community, than this Chichester case, has happened in the last half-century, and if the minority do not now act with prompt vigour and sustained activity, they will have only themselves to blame when the day of destruction comes, as it most certainly must otherwise come, to this city of imbecility and criminal neglect. It is pleasant to be able to point to one circumstance reflecting the greatest credit on the medical practitioners of Chichester. With the solitary exception above referred to the doctors have nobly stood to their guns, and, at the risk of much personal odium, have warned the inhabitants against the dangers they were perpetuating, whilst the whole of the local papers have backed them up in their manful fight against ignorance and the local ambitions of a few men, which have reduced the beautiful old cathedral city to this alarming and dangerous condition.

**Sir James Whitehead, Bart.**

Few Lord Mayors have worked harder than Sir James Whitehead, who has given so much time and energy to social questions of various kinds. Early and late the late Lord Mayor devoted himself to the task of making the office of chief magistrate of the City of London a great power for good to the people. At the outset of his year of office he presided at a meeting at St. Bartholomew's Hospital, at which Sir Sydney Waterlow read an interesting paper on the Hospital Sunday Fund. This fund has prospered greatly during his mayoralty, and at the close of its financial year on the 31st ult. the accounts showed that £41,745 had been received—that is, an excess of £1,137 over any previous year. This is the more satisfactory as no legacy or large donation has been paid to the credit of the fund during the current year. The steady but slow growth of the Hospital Sunday Fund in London is very satisfactory, and should encourage the press, to whose efforts much of the success is due, to persevere in its behalf until at least £50,000 is secured from this source for the hospitals. Mr. Henry N. Custance, the secretary, must be highly gratified by this progressive success, as he has given his best energies for many years to the Hospital Sunday movement, which has steadily advanced under his management. Sir James Whitehead has also greatly aided the Hospital Saturday Fund, and is, we understand, quite satisfied with the progress made with the penny a-week collections, which have been reorganised and extended under his personal direction. In this work he has had the devoted assistance of Mr. Bunn, the chairman of the Hospital Saturday Council. It is too early to judge how far the sum raised will be increased by this new departure, but there should be no difficulty in obtaining £50,000 a year from the working classes for the London hospitals. The proper equipment of our volunteers, the improvement of fruit culture, the encouragement of artisans by sending some of their class to the Paris Exhibition to study and report—a happy idea, by-the-bye, of Mr. Soulsby, the able private secretary to many lord mayors—and several other movements of considerable public utility have been greatly assisted by the late Lord Mayor. Indeed, the seed sown by him in many directions must bear good fruit in years to come. Altogether, it may be said that few lord mayors have better earned a baronetcy, and, in thanking Sir James Whitehead and his wife for what they have done for the charities, we heartily congratulate them both on the honour her Majesty the Queen has conferred upon them. We earnestly hope that the interests which have been awakened in Sir James Whitehead during his year of office may deepen and increase so that the medical charities, and the social movements he has become associated with, may continue to have the benefit of a large portion of his time and energies. We know what a good friend the hospitals have in the new Lord Mayor, Sir Henry Isaacs, and it is quite possible that under his guidance the Hospital Sunday Fund may reach £50,000 in 1890. May this, indeed, prove to be the case.

